# Biological foundations of successful bacteriophage therapy

**DOI:** 10.15252/emmm.202012435

**Published:** 2022-05-27

**Authors:** Carola Venturini, Aleksandra Petrovic Fabijan, Alicia Fajardo Lubian, Stefanie Barbirz, Jonathan Iredell

**Affiliations:** ^1^ Centre for Infectious Diseases and Microbiology Westmead Institute for Medical Research Westmead NSW Australia; ^2^ Faculty of Science Sydney School of Veterinary Science The University of Sydney Sydney NSW Australia; ^3^ Faculty of Health and Medicine School of Medicine Sydney Medical School The University of Sydney Sydney NSW Australia; ^4^ Department of Medicine Science Faculty MSB Medical School Berlin Berlin Germany; ^5^ Westmead Hospital Western Sydney Local Health District Westmead NSW Australia

**Keywords:** antimicrobials, bacteriophages, phage therapy, phage–bacterium dynamics, Microbiology, Virology & Host Pathogen Interaction

## Abstract

Bacteriophages (phages) are selective viral predators of bacteria. Abundant and ubiquitous in nature, phages can be used to treat bacterial infections (phage therapy), including refractory infections and those resistant to antibiotics. However, despite an abundance of anecdotal evidence of efficacy, significant hurdles remain before routine implementation of phage therapy into medical practice, including a dearth of robust clinical trial data. Phage–bacterium interactions are complex and diverse, characterized by co‐evolution trajectories that are significantly influenced by the environments in which they occur (mammalian body sites, water, soil, etc.). An understanding of the molecular mechanisms underpinning these dynamics is essential for successful clinical translation. This review aims to cover key aspects of bacterium–phage interactions that affect bacterial killing by describing the most relevant published literature and detailing the current knowledge gaps most likely to influence therapeutic success.

## Graphical abstract

This comprehensive review discusses bacterium–phage interactions, antimicrobial potential of phages and research challenges that impact realization of the successful bacteriophage therapy.

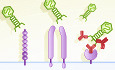

**Glossary**
Bacteriophages: Bacteriophages, or phages, are viruses that specifically and selectively infect bacteria
Biofilm: Surface‐attached, structured community of microorganisms embedded in a self‐produced extracellular matrix (polysaccharides, DNA, water)
Enzybiotics: Phage‐derived antibacterial enzymes with therapeutic potential. Depolymerases catalyse the hydrolysis of the capsule polysaccharide of Gram‐negative bacteria, while lysins (also endolysins or murein hydrolases) are hydrolytic enzymes capable of cleaving the cell wall (peptidoglycan) of both Gram‐negative and Gram‐positive species
L‐forms: Cell wall‐deficient bacteria resistant to supra‐therapeutic concentrations of cell wall targeting antibiotics
Lysogenic conversion: Phage–bacterium interaction in which a prophage encodes proteins that enhance bacterial fitness or virulence
Lysogeny or lysogenic cycle: Phage life cycle in which the viral genome stably integrates in the bacterial chromosome, replicating with it
Lytic infection and productive lysis: Infecting phages replicate their genome and assemble new viral particles (virions) by hijacking host resources. Phage‐directed cell lysis then releases this viral progeny ready to infect new cells, in an exponential growth cycle (productive lysis) limited only by availability of bacterial prey and their response/s to phage attack
Obligate lytic phages: Phages that cannot undergo lysogeny. Preferred for therapeutic applications
Phage adsorption: Molecular interactions between phage proteins and specific bacterial receptors that bind the phage to the bacterial cell surface allowing for infection (phage genome release into the cytosol) to occur
Phage cocktail: Combination of multiple phages for therapeutic application. Phages in a cocktail ideally act synergistically against a bacterial target and limit the development of phage‐resistant variants. Cocktails combining phages with different host specificity allow for broader therapeutic targeting
Phage therapy: Medical use of phages as antimicrobials for treatment of bacterial infections
Pseudolysogeny: Phage–bacterium interaction in which the phage genome resides within the host cell without chromosomal integration, in an unstable, inactive state
Temperate phages: Phages capable of undergoing lysogeny. These may lie “dormant” within a living bacterial cell while integrated into the host chromosome as “prophages”, but have the potential to enter a lytic infection cycle under certain conditions (e.g. host cell stress). Temperate phages are less preferred for therapy
Transduction: Phage‐mediated horizontal transmission of genetic information from one bacterial cell to another, as opposed to genetic inheritance through reproduction (“vertical transmission”). Mainly associated with the lysogenic life cycle

## Brief introduction to phage therapy

With the discovery of antibiotics and the development of vaccines, the 20^th^ century saw an unprecedented steady decline in mortality attributable to bacterial infections (Armstrong *et al*, 1999). This progress built on advances in microbiology and sanitation in the 1880s led by Louis Pasteur and Ignaz Semmelweis (Cavaillon & Chrétien, 2019). In the late 1910s, following initial work by Ernest Hankin and Frederick Twort, Felix D’Herelle identified viruses that specifically and selectively kill bacteria, naming them bacteriophages (phages) [from “bacterium” + “phagêin” (*Greek*, to eat)], and immediately recognized their potential as antimicrobial agents (Sulakvelidze *et al*, 2001; Kutter & Sulakvelidze, 2004; Wittebole *et al*, 2014). In the following decades, however, the development of phage‐based therapy was hampered by a poor understanding of phage biology, some early clinical failures and the meteoric rise of antibiotics (Sulakvelidze *et al*, 2001; Wittebole *et al*, 2014; Rohwer & Segall, 2015).

Regrettably, the use (and misuse) of antibiotics has since led to the emergence of globally disseminated bacterial pathogens that are resistant to last‐line treatments, and antibiotic resistance now poses a significant global health and economic burden (Fair & Tor, 2014; O'Neill, 2016; WHO, 2017; Baker *et al*, 2018). As investment in the discovery and production of new antibiotics dwindles, the development of alternative antimicrobial therapies, including revaluation of phage therapy, is a primary goal (Moelling *et al*, 2018; Rohde *et al*, 2018; Petrovic Fabijan *et al*, 2020a).

In parts of eastern Europe (e.g. Georgia, Poland and Russia), phages have been in routine medical practice for over 70 years and this experience provides a rich source of empirical data (Sulakvelidze *et al*, 2001; Stone, 2002; Rohwer & Segall, 2015; Górski *et al*, 2020). Several reviews of recent progress in the development of phage therapy cover preclinical experimentation in animal models, compassionate use in critically ill humans and a few clinical trials (Wittebole *et al*, 2014; McCallin & Brüssow, 2017; Gordillo Altamirano & Barr, 2019; Nale & Clokie, 2021; Pirnay & Kutter, 2021). Most of the cited studies attest to the safety of phage therapy, but clinical effectiveness has not yet been conclusively demonstrated (McCallin & Brüssow, 2017; Gordillo Altamirano & Barr, 2019; Pirnay & Kutter, 2021). In addition, the results of experimentation in small animal models do not consistently translate into clinical success (Wittebole *et al*, 2014; Nale & Clokie, 2021), just as *in vitro* phage activity often fails to correlate with *in vivo* efficacy (Melo *et al*, 2020a). These inconsistencies complicate the design of clinical protocols, undermine confidence in phage application and hinder progress towards clinical implementation.

**Figure 1:**
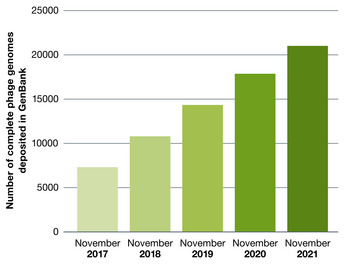
Phage whole genome sequencing Number of complete phage genomes deposited in GenBank in the past 5 years (with permission from Cook *et al*, 2021).

While the number of completely sequenced phage genomes has doubled in the last 5 years (Fig 1) (Cook *et al*, 2021), these represent a minuscule fraction of the prokaryotic virosphere, estimated to exceed 10^31^ particles (Hatfull, 2015). Phages are found in all bacterial habitats (Kutter & Sulakvelidze, 2004; Clokie *et al*, 2011) and are a key driving force of microbial ecology and evolution (Dion *et al*, 2020). Tailed double‐stranded DNA phages (order *Caudovirales*) constitute the largest group described to date (Clokie *et al*, 2011) and are easily isolated with simple techniques from diverse environmental sources (Ackermann, 1998). Tailed phages have high target specificity, which can be redirected by forced evolution or genetic engineering (Pires *et al*, 2016a; Burrowes *et al*, 2019), and are the only phage type to have been trialled in therapy so far (Ackermann, 1998; Kutter & Sulakvelidze, 2004).

Phages eliminate target bacteria by bursting bacterial cells (lysis) within minutes of infection (Kutter & Sulakvelidze, 2004), thereby releasing newly formed phage particles (virions) that go on to infect new host cells in a self‐perpetuating cycle (Kutter & Sulakvelidze, 2004; Kortright *et al*, 2019). Crucially, phage activity is unaffected by antibiotic resistance.

The highly specific virus–host pairing is central to microbial population dynamics and is deeply connected to environmental conditions and ecological niches. In therapeutic applications, the risk of undesirable adaptive outcomes of the phage–bacterium interplay (e.g. resistance development in bacteria) is pragmatically addressed by the use of combinations of multiple phages (cocktails) with differing adaptive strategies (Chan & Abedon, 2012; Chan *et al*, 2013; Pirnay *et al*, 2018; Rohde *et al*, 2018). Combining phages into therapeutic cocktails (as opposed to monophage therapy), broadening their utility and commercialization potential, requires a clear understanding of phage–phage and phage–bacterium dynamics (Chan & Abedon, 2012; Schmerer *et al*, 2014; Gordillo Altamirano & Barr, 2019; Venturini *et al*, 2019a; Pirnay, 2020; Haines *et al*, 2021).

The key mechanisms that may allow prediction of *in vivo* pharmacokinetics and dynamics linked to therapeutic outcome have not yet been fully elucidated. Here, we provide an overview of the biological processes linked to phages’ antimicrobial potential and highlight some of the research challenges that remain.

## Phage infection

### Infection cycles

Phages depend on their bacterial hosts for survival and multiplication, but bacterial growth rates can fluctuate significantly even in nutritious habitats. Doubling times for wild‐type *Escherichia coli* laboratory strains in optimal conditions are approximately 20 min (Gibson *et al*, 2018), while those measured in the mammalian gut can range from 40 min to 140 h (Abedon, 1989; Poulsen *et al*, 1995). Although one infective phage particle may yield as many as 20,000 new virions per infected bacterial cell in optimal conditions (Zinder, 1980), bacteria rarely encounter such habitats in nature and phages that would ordinarily propagate exponentially may fail to do so when bacterial growth is limited (e.g. by nutritional stress) (Lourenço *et al*, 2020; Attrill *et al*, 2021).

**Figure 2:**
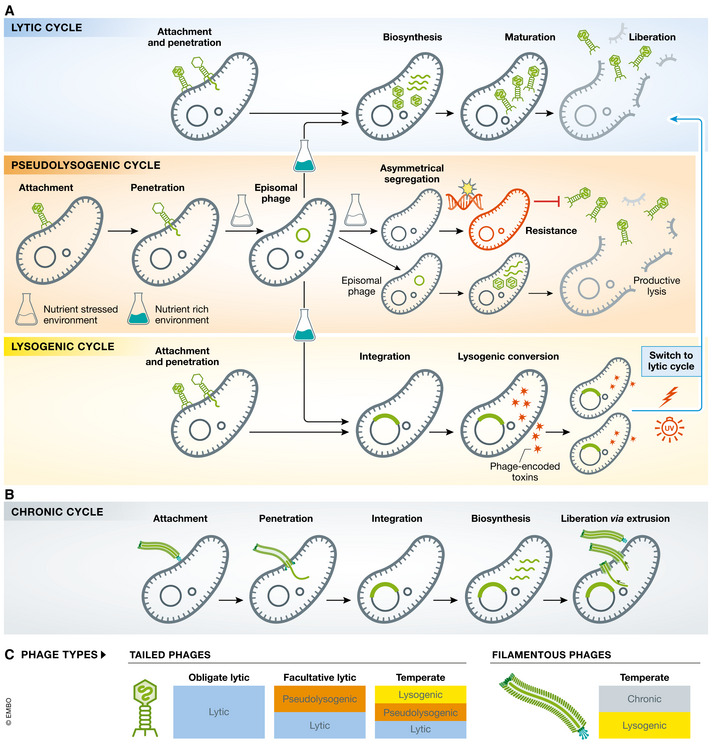
Phage replicative cycles (A) Modes of phage infection characteristic of tailed phages: (i) *lytic cycle*—phage replication immediately follows infection, with assembly and release of virions leading to cell lysis. Each virion is free to start a new lytic cycle leading to a burst of “productive” infection; (ii) *lysogenic cycle*—phages can integrate into the bacterial chromosome and replicate with it as prophages, until a lytic cycle is triggered; and (iii) *pseudolysogeny*—phage genomes persist in a episomal state within the host cell before resolving into a lytic or lysogenic cycle. Episomal phages typically segregate asymmetrically during cell division, while a small fraction undergoes a productive lytic cycle (scavenger response) favouring development of phage‐resistant bacterial subpopulations. (B) Chronic infection cycles are characteristic of “filamentous phages” (family *Inoviridae*) that continuously produce progeny released by extrusion without cell death/lysis. (C) Phage types by replication cycle: tailed phages that always lyse bacteria upon infection are “virulent” or “obligate lytic”, while “facultative lytic” phages may also undergo pseudolysogeny. “Temperate” phages may have a lysogenic or pseudolysogenic lifestyle until triggered to enter a lytic cycle, typically when the host bacteria experience stress conditions. Filamentous phages typically follow a chronic productive cycle, though some have the capacity to also enter a lysogenic cycle.

In exponentially growing bacteria, phages replicate typically via either a lytic or a lysogenic cycle (Fig 2A). Phage therapy traditionally uses “virulent” or “obligate lytic” phages (lytic cycle only) that lyse bacteria immediately upon infection in preference to “temperate” phages, which undergo a lysogenic cycle, integrating their genome into the bacterial host chromosome and replicating passively with it as “prophages” (Fig 2A–C) (Lamont *et al*, 1989; Howard‐Varona *et al*, 2017; Li *et al*, 2020). Therapeutic use of temperate phages risks transfer of genes (“transduction”) that may enhance bacterial fitness or virulence (e.g. toxins) or confer antibiotic resistance to the bacterial host (Brussow *et al*, 2004). This is known as “lysogenic conversion”, a process by which important pathogens have acquired cardinal virulence factors (e.g. *Corynebacterium diphtheriae* carrying the siphovirus β‐phage that encodes the diphtheria toxin Tox (Holmes, 2000) or enterohaemorrhagic *E. coli* with the lambdoid phage encoding Shiga toxins (Schmidt, 2001)). Stable chromosomal integration is mainly a function of the phage itself (Brussow *et al*, 2004; Fortier & Sekulovic, 2013; Argov *et al*, 2019; Petrovic Fabijan *et al*, 2021) but also depends on host conditions; when these change (e.g. nutritional stress or DNA damage), prophages may excise from the chromosome and enter a lytic cycle that leads to bacterial cell death (Banks *et al*, 2003; Nanda *et al*, 2014; Balasubramanian *et al*, 2019; Chatterjee & Duerkop, 2019; Benler & Koonin, 2020; Filipiak *et al*, 2020). Importantly, quorum‐sensing mechanisms and communication via signalling molecules are also increasingly implicated in phage–bacterium interactions, including switching between lytic and lysogenic lifestyles (León & Bastías, 2015; Silpe & Bassler, 2019).

Chronic infection is a distinct replication cycle characteristic of “filamentous” phages belonging to the family *Inoviridae* (single‐stranded DNA phages; order *Tubulavirales*) (Fig 2B) (Secor *et al*, 2020; Mantynen *et al*, 2021). Unlike lytic and lysogenic cycles, chronic infection leads to continuous virion production without lysis of the bacterial cell (Loh *et al*, 2019). Filamentous phages are well suited for the horizontal exchange of DNA and many encode important virulence factors. The best understood filamentous phages involved in lysogenic conversion of their hosts are those that infect *Vibrio cholerae* (e.g. CTXø, encoding the cholera toxin) (Waldor & Mekalanos, 1996; Karaolis *et al*, 1999) and Pf phages that infect *Pseudomonas aeruginosa* promoting biofilm production in infected bacteria (Secor *et al*, 2015). Filamentous phages are considered unsuitable for therapy.

While lytic and lysogenic lifestyles and their impact on therapeutic outcome have been extensively reviewed (Sulakvelidze *et al*, 2001), the impact of pseudolysogeny has not yet been defined. This additional phage infection mode (Fig 2A), which some propose should be defined altogether as a separate cycle (Mantynen *et al*, 2021), was first recognized in the early 1960s (Los & Wegrzyn, 2012), but as yet there is no unanimous definition for this phenomenon and its molecular bases remain largely unexplored. Pseudolysogeny has been defined as a “phage carrier” state (Ripp & Miller, 1997) or, perhaps more accurately, as “stalled phage development” (Los & Wegrzyn, 2012). In pseudolysogeny, neither multiplication nor synchronized replication of the phage occurs within the host cell, but when conditions allow, the phage enters either a “normal” lysogenic cycle or a lytic cycle.

Pseudolysogeny has been observed primarily in Gram‐negative species, generally when bacterial growth was limited (Los *et al*, 2003; Cenens *et al*, 2013; Latino *et al*, 2016), suggesting a role in long‐term phage survival in unfavourable conditions, perhaps by providing many of the advantages of the lysogenic state while avoiding chromosomal integration. Lytic phages are recognized by their efficient killing activity *in vitro* (high lytic efficacy) and the absence of classic lysogeny genes (integrases, repressor genes, etc.), but there is no established genetic marker of pseudolysogenic capacity, as it is not usually a feature of exponentially growing bacteria. Replication of obligate lytic T4‐like phages is completely inhibited in nutrient‐stressed *E. coli*, but it has been reported that under the same conditions, a T4rI mutant (defective in the function of the holin inhibitor) keeps producing viable virions (Los *et al*, 2003). Bryan *et al* (2016) showed that T4 phages efficiently bind to and infect, but fail to successfully lyse, *E. coli* in the stationary phase. Under nutrient‐limiting conditions *in vitro*, the lytic cycle still occurs in a small subpopulation of infected bacterial cells (“scavenger response”), fully resuming in the rest of the population only upon nutrient addition with restoration of logarithmic growth. *P*. *aeruginosa* and *Yersinia enterocolitica* can support pseudolysogenic infection with apparently obligate lytic myoviruses or podoviruses that provide bacteria with immunity from further phage infection (superinfection exclusion) (Latino *et al*, 2016; León‐Velarde *et al*, 2016). Thus, it seems that not all virulent phages are truly obligate lytic viruses or, at least, that a replication pause may occur in the lytic cycle. This provides advantages for both the virus and the parasitized host cell, especially when the host bacterial population is stressed, by preventing extinction of vulnerable bacterial population on which the predating virus is dependent.

Temperate phages may also enter a pseudolysogenic state in bacteria that are stressed or starved: the podovirus P22 can stably persist in episomal form in *Salmonella* cells, asymmetrically segregating upon cell division (Cenens *et al*, 2013). This is linked to the specific phage‐mediated and targeted depression of the host *dgo* operon via the *pid* phage gene (Cenens *et al*, 2013) and suggests some advantages of pseudolysogeny even in phages that have developed the capacity to integrate, perhaps as a more agile response to bacterial population stress. Other temperate phages, variably defined as “phage‐like plasmids” (Pfeifer *et al*, 2021) or “phagemids” (Kittleson *et al*, 2012), are found in the host as extra‐chromosomal elements that encode partitioning systems (Salje, 2010) and replicate within the cell cycle. In the well‐studied P1 *E. coli* myovirus (Lobocka *et al*, 2004) and its many variants (Walker & Anderson, 1970; Rosner, 1972; Venturini *et al*, 2019b), ATP‐dependent post‐segregational killing promotes symmetrical distribution of phage episomes via common plasmid partitioning and maintenance mechanisms (Lobocka *et al*, 2004).

Although much remains to be investigated, it seems plausible for pseudolysogeny to represent a route to both short‐ and long‐term phage survival through (i) physical protection from harsh environmental conditions outside the host (e.g. UV‐light, pH and temperature can drastically reduce the half‐life of virions) (Jonczyk *et al*, 2011), and (ii) hibernation (replication pause) in unfavourable conditions that threaten the host population (e.g. stationary phase or persister populations) (Bryan *et al*, 2016).

A better understanding of the diversity and genetic regulation of phage life cycles is paramount for successful therapeutic applications. Future progress will likely benefit from “multiomics” approaches and investigation of these complex phenomena at a single‐cell level (Dang & Sullivan, 2014; Skurnik, 2022). Genetic engineering approaches may also prove useful for redirecting phage lifestyles to suit therapeutic goals (e.g. enhance lysis by elimination of lysogeny genes in temperate phages (Dedrick *et al*, 2019)).

### Multiplicity of infection and the concept of phage dosing

Self‐amplification through progressive productive infection is a unique distinction between phages and traditional (drug) antibiotics with important clinical implications (Levin & Bull, 2004). Phage growth parameters such as adsorption rate, latent period (duration of infection cycle from replication to virion assembly) and burst size (number of released virions per lysed cell) are commonly used to quantify productive lytic infection *in vitro* (Levin & Bull, 2004; Dennehy & Abedon, 2021). These parameters are specific to each phage and can vary considerably, and as such have been the focus of theoretical studies attempting to model lysis outcomes of bacterium–phage pairs to inform therapeutic strategies (Bull *et al*, 2004; Levin & Bull, 2004; Wang, 2006; Heineman & Bull, 2007).

Modelling of *in vivo* dynamics, even for the simplest phage–bacterium interaction, must consider the availability of resources to bacterial prey populations (Weitz *et al*, 2013), other mobile genetic elements (Harrison *et al*, 2017), community effects (bystander microflora) (Blazanin & Turner, 2021) and the spatial structures at the site where predator and prey meet (Lourenço *et al*, 2020; Attrill *et al*, 2021). Bacterial density directly affects adsorption rate and phage replication duration, as well as opportunities for further viral propagation. If target bacteria are slow‐growing and sparsely separated, the productive exponential infection may not proceed (Payne & Jansen, 2001; Kasman *et al*, 2002; Levin & Bull, 2004; Heineman & Bull, 2007; Abedon, 2011).

Multiplicity of infection (MOI) is the term used to indicate the ratio of phages to bacteria in *in vitro* testing and is often applied *in vivo* as a “dosing” concept. A MOI of > 10 may be more advantageous in murine sepsis models (Yuan *et al*, 2019; Hesse *et al*, 2020), and this has been used as a target for human dosing (Khatami *et al*, 2021), but this extrapolation is problematic because not all phages administered reach their target and not all phages that adsorb to a bacterial cell will infect it (Attachment mechanisms and receptor specificity) (Abedon, 2016). Direct measurement of phage and bacterial densities *in vivo* is not practical except for urine (Abedon, 1989; Khawaldeh *et al*, 2011; Dąbrowska & Abedon, 2019) or blood (Petrovic Fabijan *et al*, 2020b) so that even once the target MOI is defined and the amplification process can be monitored, these samples of convenience can only serve as surrogates for the site of infection in tissues. Therapy with antibiotic drugs leads to relatively predictable relationships between tissue and blood concentrations, which can be determined and used to optimize dosing. Evidence of phage amplification derived from samples of convenience might become a useful surrogate for successful delivery to site. However, *in vivo* amplification appears to subside quickly, likely due to both therapeutic “success” (i.e. elimination of prey populations) and host control of the administered therapeutic virus by innate and acquired immune responses (The eukaryotic host: phage‐induced immune responses). The pharmacodynamics and pharmacokinetics of phage therapy are also subject to variable and possibly virus‐specific tissue penetration (Górski *et al*, 2015; Dąbrowska & Abedon, 2019). Careful monitoring of clinical sites and samples in the course of carefully structured therapeutic regimens will therefore be extremely important to lasting and robust therapeutic application (Abedon, 2011).

### Attachment mechanisms and receptor specificity

Phage adsorption to the bacterial cell is a first and crucial step in the infection process (Bertozzi Silva *et al*, 2016; Letarov & Kulikov, 2017). For “best” phage therapy (optimal lytic efficiency = optimal bactericidal activity), the majority of virus–bacterium contacts should lead to productive infection (Multiplicity of infection and the concept of phage dosing), making the molecular interactions at the bacterial cell surface a key aspect of therapeutic success (Nobrega *et al*, 2018). Membrane‐embedded proteins are common phage receptors, but phage access to these receptors is highly regulated by various protective glycan structures such as peptidoglycan, capsule or lipopolysaccharide (LPS) found on bacterial envelopes.

#### Phage tail machines as sophisticated infection devices

**Figure 3:**
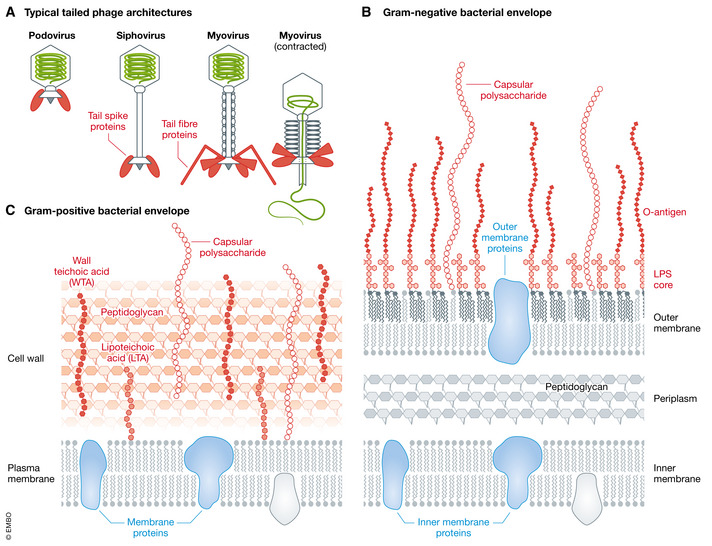
Bacterial envelopes and receptors for tailed phages (A) Schematic overview of the three typical tailed phage architectures. Fibre and spike components in the baseplate that interact with bacterial envelope glycans are shown in red. (B) Gram‐negative bacterial envelope. (C) Gram‐positive bacterial envelope with glycan (red) and protein (blue) phage receptors.

Although phage–bacterium interactions via capsid proteins have been described (Casjens & Molineux, 2012), adsorption to the bacterial cell envelope is most commonly mediated by sophisticated phage tail machines that specifically recognize diverse bacterial cell surface structures and are implicated in other important infection‐aiding processes (Chua *et al*, 1999; Letarov & Kulikov, 2017; Nobrega *et al*, 2018) (Fig 3). Three tail morphologies are known: short non‐contractile tails in the *Podoviridae*; long non‐contractile tails in the *Siphoviridae*; and long contractile tails in the *Myoviridae* (Ackermann & Prangishvili, 2012) (Fig 3A). Tailed phages have evolved to deliver much larger genomes to their hosts than non‐tailed phages (Davidson *et al*, 2012) and are highly specialized in overcoming the protective layers of Gram‐negative and Gram‐positive bacterial envelope architectures (Fig 3B and C). For host recognition, tailed phages use fibres, longitudinal, multimeric protein assemblies, or shorter and more compact protein oligomers termed spikes.

#### Surface attachment and infection

Phage recognition of bacterial cell surface receptors is a well‐orchestrated process comprising several individual sequential steps (Broeker & Barbirz, 2017; Broeker *et al*, 2017). The diversity of bacterial cell envelopes (Fig 3) has required tailed phages to develop different strategies to initiate infection. This initial and often reversible interaction with the primary receptors precedes subsequent “secondary receptor” binding, which leads to changes in the tail machine that are irreversible (Casjens & Molineux, 2012). Phages preferentially encounter as primary receptors all the exposed surface structures on host bacteria, *i.e*. capsule, exopolysaccharide, peptidoglycan or teichoic acids (Dunne *et al*, 2018), and in Gram‐negative target also LPS (Broeker & Barbirz, 2017). Flagella and adhesins may also serve as primary receptors for some phages (Esteves *et al*, 2021; Montemayor *et al*, 2021). Many primary receptors are distal to the cell surface, and phages employ diverse active mechanisms to approach the bacterial membrane. “Flagellotropic” phages, for example, ride on flagella towards the host surface, harnessing bacterial motility for infection progression (Tittes *et al*, 2021), and many tailed phages produce tailborne depolymerases to specifically destroy the polysaccharide‐based glycan protective shields (Knecht *et al*, 2020). Many of the initial fibre‐ or spike‐mediated receptor interactions are reversible, which allows phage particles to dissociate from the cell surface until they reach a site for irreversible attachment.

Irreversible attachment to these secondary receptors can initiate a cascade of steps that lead to permanent conformational rearrangements in the phage tail assembly (Taylor *et al*, 2018), priming the phage for DNA release. Conserved transmembrane proteins (e.g. transporters and channels), efflux pumps and pilus portals often serve as secondary receptors (Bertozzi Silva *et al*, 2016), with their extracellularly exposed parts providing highly specific phage attachment sites with numerous opportunities for bacterial adaptation to halt the phage infection cycle, for example by mutation of outer membrane extracellular loops (Porcek & Parent, 2015; Rocker *et al*, 2020). As shown for purified outer membrane proteins (Chiaruttini *et al*, 2010; Evilevitch, 2018), binding to secondary receptors can trigger the phage molecular machine for DNA release *in vitro*, thus rendering phage particles non‐infectious. Gram‐negative host‐specific phages incubated with protein‐free LPS preparations typically lose their infectivity as contact with these receptor molecules induces particle opening and DNA loss (Jesaitis & Goebel, 1955; Lindberg, 1973; Andres *et al*, 2012; Broeker *et al*, 2019). How entirely protein‐free LPS preparations trigger DNA release in the absence of a host cell remains to be elucidated (Andres *et al*, 2010; Broeker & Barbirz, 2017). Cryotomography studies of phages attached to bacteria have revealed the formation of channel‐like structures spanning the envelope that ensure the integrity of the cell surface during phage genome transfer into the cytosol. However, the molecular composition of these channels is not yet fully understood (Hu *et al*, 2015; Farley *et al*, 2017; Wang *et al*, 2019); in some cases, phages seem to extend their tails to reach the interior of the cell, while in others, phage‐synthesized ejection proteins recruit other protein components from the bacterial envelope to facilitate DNA movement.

#### Adsorption regulation—the unique role of bacterial surface glycans

In bacterial ecosystems, regulation of interactions with predatory viruses takes place both at extracellular and at intracellular levels (Hampton *et al*, 2020). As phage receptors, surface glycans (Fig 3) modulate phage entry and are important in evolutionary adaptations to phage infection (Phage–bacterium co‐adaptation). Bacterial cell surface glycans also face the human immune system and are often described as important participants in so‐called pathogen‐associated molecular patterns (PAMPs). Changes induced by phages thus affect the innate immune response to pathogens (The eukaryotic host: phage‐induced immune responses), and phage‐encoded enzymes that remove protective glycan layers (e.g. depolymerases), exposing underlying PAMPs at the envelope (Majkowska‐Skrobek *et al*, 2018; Liu *et al*, 2020; Volozhantsev *et al*, 2020), may directly enhance clearance of bacteria by the innate immune system (Oliveira *et al*, 2019a).

In the presence of actively infecting phages, bacteria may alter surface glycan structures through transcriptional control of glycosyltransferases. This “phase variation” is achieved by altered glycan composition or LPS chain length or even by complete abrogation of the assembly of protective capsule or O‐antigens (Huan *et al*, 1997; Seed *et al*, 2012; Cai *et al*, 2019; de Sousa *et al*, 2020; Whitfield *et al*, 2020). Similarly, prophages may alter bacterial surface glycan composition via glycosylation or acetylation to exclude other phages from infection (Cenens *et al*, 2015; Schmidt *et al*, 2016; Teh *et al*, 2020).

Phages bind bacterial surface glycans using specific tail proteins (Broeker *et al*, 2017; Nobrega *et al*, 2018; Knecht *et al*, 2020). Many host adsorption proteins are depolymerases that facilitate surface access through O‐antigen or capsular layers, this being an essential step for infection by some phages (Broeker & Barbirz, 2017). The glycan adsorptive capacity of these tail proteins also enables phages to persist in glycan‐rich niches, for example by binding heparan sulphates of mucins in the mammalian gut (Green *et al*, 2021). Phage glycan depolymerases can strip off glycan coats without initiating cell rupture, thereby avoiding critical concentrations of microbial cell envelope fragments that may drive a damaging immune response in clinical sepsis (Ryu *et al*, 2017). LPS‐mediated sepsis and septic shock are primary drivers of mortality in Gram‐negative infection (Opal *et al*, 1999), and several studies have shown that pretreatment with phage depolymerases to degrade O‐antigen polysaccharides reduces pro‐inflammatory responses and protects mice from lethal sepsis (Liu *et al*, 2019; Oliveira *et al*, 2019b; Chen *et al*, 2020).

Outer membrane vesicles (OMVs) also play a unique role in controlling phages as they can effectively trap them, preventing host infection (Schwechheimer & Kuehn, 2015; Reyes‐Robles *et al*, 2018), as shown for *Salmonella* phage P22 where only few phages eject their DNA into the OMV lumen, with the majority of particles stalling at the membrane (Stephan *et al*, 2020).

The specificity of these attachment mechanisms limits phage clinical range, when compared to traditional antibiotics with broad‐spectrum activity against multiple bacterial species. This potential therapeutic limitation is mainly being obviated by the use of phage cocktails, but it can also be addressed via natural phage “training” to broaden host range by successive passage (Yu *et al*, 2015; Burrowes *et al*, 2019) or by formal synthetic biology approaches (Chen *et al*, 2019; Dedrick *et al*, 2019). The use of enzybiotics (depolymerases or endolysins) is also being considered (Pires *et al*, 2016b; Olsen *et al*, 2018). Phage endolysins attack the peptidoglycan layer of Gram‐positive and Gram‐negative envelopes (Fig 3), thus acting less specifically than depolymerases (Broendum *et al*, 2018; Sao‐Jose, 2018; De Maesschalck *et al*, 2020; Mondal *et al*, 2020; Chen *et al*, 2021; Linden *et al*, 2021; Murray *et al*, 2021). Importantly, the development of bacterial resistance to externally applied endolysins is unlikely as these enzymes target cellular structures essential for bacterial survival (Roach & Donovan, 2015). However, all the outlined approaches crucially require the maintenance and accessibility of well‐curated and diverse phage banks, which are still scarce (Nagel *et al*, 2022).

### Phage–bacterium co‐adaptation

The interaction between phages and bacteria is a major contributor to the diversity and evolution of microbial populations, involving fine‐tuned, complex co‐adaptation dynamics, with bacteria trying to minimize susceptibility to phage infection as phages strive to retain or regain it (Díaz‐Muñoz & Koskella, 2014; Koskella & Brockhurst, 2014; Seed *et al*, 2014). Bacterial adaptations are not without cost, and both mathematical models and experimental observations suggest that bacterial resistance to phage can be overcome (Levin & Bull, 2004), but the development of bacterial phage resistance *in viv*o has not been yet systematically researched (Hesse *et al*, 2020; Gordillo Altamirano *et al*, 2021; Salazar *et al*, 2021).

Alteration of cell surface phage receptors (“adsorption resistance”, through modification or masking or by synthesis of competitive inhibitors; Attachment mechanisms and receptor specificity) is arguably the most common adaptive response to phage predation; CRISPR/Cas may be a close second (Doron *et al*, 2018; Ofir & Sorek, 2018; Alseth *et al*, 2019; Rostøl & Marraffini, 2019; Hampton *et al*, 2020). Bacterial susceptibility to phages may be modulated by horizontal exchange of receptors mediated by OMVs (Tzipilevich *et al*, 2017) or more often by genetic modification of cell surface structures targeted by phages, which may affect both pathogenic potential and overall survival of target bacteria (Verma *et al*, 2009; Capparelli *et al*, 2010; Chan *et al*, 2016; Markwitz *et al*, 2021). The resulting fitness cost can increase bacterial vulnerability to both the immune system and antibiotics (León & Bastías, 2015).

Attempts to use phages to clear *Klebsiella pneumoniae* and *Acinetobacter baumannii* infection *in vivo* have resulted in phage‐resistant capsular mutants that appear to be less virulent and more susceptible to antibiotics (Verma *et al*, 2009; Gordillo Altamirano *et al*, 2021), and therefore easier to eliminate. *E. coli* responds to phage challenge by modification of LPS biosynthesis with concomitant fitness loss and attenuation in a murine model of systemic infection (Salazar *et al*, 2021). In *K. pneumoniae*, mutations in the porin OmpK36 lead to increased antibiotic resistance and are poorly tolerated *in vivo* (reduced growth rates) (Fajardo‐Lubian *et al*, 2019), while in *Shigella flexneri* Omp‐targeting phages have been shown to lead to resistant mutants incapable of intracellular spread (Kortright *et al*, 2022). As such, Omp‐specific phages, for example, might have particular value in managing these pathogens. Phage‐insensitive variants appear to be rarely isolated after phage administration in the clinic, suggesting that the many varied outcomes predicted and observed *in vitro* may be transient *in vivo*, with few phage‐resistant subtypes (“fittest” mutants) actually able to succeed in nature (Bohannan & Lenski, 2000; León & Bastías, 2015; Hernandez & Koskella, 2019; Aslam *et al*, 2020; Petrovic Fabijan *et al*, 2020b).

Conversely, phages may counterevolve to regain their infectivity by modification of their own host attachment receptors (tails), resulting in host range expansion (Salazar *et al*, 2021). In therapy, the use of cocktails of multiple phages acting in synergy (to optimize lysis of target bacteria) has been shown to both broaden target range and minimize the occurrence of phage resistance (Abedon *et al*, 2021a). While the development of cross‐resistance is also a possibility (Wright *et al*, 2018), mixtures of phages with different receptor specificities are expected to exert multiple simultaneous selective pressures on the target host (Schmerer *et al*, 2014) that come at increased costs to bacterial fitness. Carefully “tailored” phage combinations using original and “evolved” phages against the one host have been shown to successfully target both the wild‐type strain and its variants (Yu *et al*, 2018; Aslam *et al*, 2020; Abedon *et al*, 2021a; Salazar *et al*, 2021).

Phage attack can affect antibiotic susceptibility in target bacteria (Ryan *et al*, 2012; Segall *et al*, 2019; Gordillo Altamirano *et al*, 2021), and the careful use of antibiotic–phage combinations may also be useful in limiting the development of bacterial variants resistant to both (Gu Liu *et al*, 2020 *et al*, 2020; Gordillo Altamirano *et al*, 2021). As outlined in several recent exhaustive reviews (Segall *et al*, 2019; Tagliaferri *et al*, 2019; Morrisette *et al*, 2020; Li *et al*, 2021), phage–antibiotic synergy (PAS) has been successfully demonstrated in both Gram‐positive and Gram‐negative bacteria, though many studies have focused on *E. coli* and *P. aeruginosa* (Comeau *et al*, 2007; Allen *et al*, 2017; Chaudhry *et al*, 2017; Gu Liu *et al*, 2020), and may have important clinical implications. However, synergy is not the only outcome of simultaneous exposure to phages and antibiotics with addition, neutrality and antagonism also possible.

The effects of phage–antibiotic combinations on target bacteria depend on many factors including the specific antibiotic tested (results obtained with one antibiotic are not always replicated with another antibiotic of the same class), the testing conditions (e.g. type of media, bacterial growth (planktonic cells versus biofilm), *in vitro* versus *in vivo* conditions), phage type (even very closely related phages can give different outcomes), and timing of administration (e.g. simultaneous or sequential) (Segall *et al*, 2019; Tagliaferri *et al*, 2019; Morrisette *et al*, 2020; Li *et al*, 2021). Only recently, Gu Liu *et al* (2020) presented the first in‐depth analysis of the mechanisms underlying the efficacy of phage–antibiotic combinations against a highly virulent *E. coli* ST131 strain. Their work clearly demonstrates the complexity of these interactions and the urgent need for applying this type of comprehensive approach to other bacterial species and antibiotic–phage combinations for a clear understanding of possible outcomes to guide clinical application.

Phage–bacterium co‐adaptation is predicted to drive a stalemate that favours bacterial survival in nature (Bohannan & Lenski, 2000; Koskella & Brockhurst, 2014; Fernández *et al*, 2018; Makalatia *et al*, 2021), and successful therapy requires us to contrive situations in which natural balances are tipped in favour of the phage (Levin & Bull, 2004), the specifics of which will depend on the interacting phage–bacterium pair and their immediate environment. Phage‐resistant variants arising *in vivo* can be problematic (Schooley *et al*, 2017), but phage‐resistant bacteria are sometimes less virulent (Olszak *et al*, 2019) or less antibiotic‐resistant (Oechslin, 2018) than their parent (Ryan *et al*, 2012; Chaudhry *et al*, 2017). A detailed understanding of receptor specificities (Bertozzi Silva *et al*, 2016) and co‐adaptation trajectories both *in vitro* and *in vivo* (Doron *et al*, 2018; Makalatia *et al*, 2021) must be developed in order to inform new mathematical models and “artificial intelligence” (AI) solutions (Schmerer *et al*, 2014; Cowley *et al*, 2018; Hesse *et al*, 2020; Pirnay, 2020; Haines *et al*, 2021; Maffei *et al*, 2021) to help deconvolute these natural biological and evolutionary complexities.

## Bacterial targets

### Reduced growth states: stationary phase bacteria and L‐forms

**Figure 4:**
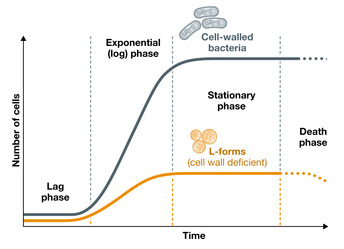
*Escherichia coli* growth states Growth in optimal conditions (37°C; rich medium) of wild‐type *E. coli* (blue curve) and its L‐forms (cell wall‐deficient variants; orange curve). At reaching stationary phase, bacterial metabolic activity and growth are slowed to near nil.

Bacterial pathogens have evolved to defend themselves effectively against commonly encountered stressors in the mammalian host (e.g. oxidative, nutritional and antibiotic). Given the ubiquity of phages in nature and the aeons of co‐evolution with bacteria, an array of finely tuned and well‐established defences against phage attack are also to be expected. The physiological state of the bacterial host population is an important determinant of phage replication (Infection cycles), and the exponential growth conditions used for antibiotic and phage susceptibility testing in diagnostic laboratories are probably rare in nature, with “stationary phase” growth being common in chronic and relapsing infections (Gefen *et al*, 2014) (Fig 4).

The impact of bacterial stress on the lytic/pseudolysogenic pathways may be therapeutically important. Phages that ordinarily pseudolysogenize stressed bacteria (Bryan *et al*, 2016) may be poor choices for the management of some infections. Cell wall‐deficient “L‐forms” are more metabolically active and faster growing than stationary phase‐walled cells (Mercier *et al*, 2014; Mickiewicz *et al*, 2019) but divide more slowly than exponential phase bacteria (Fig 4), using a primitive mechanism that is independent of essential elements of binary fission (e.g. FtsZ) (Leaver *et al*, 2009). L‐forms can be induced by innate immune effectors, such as lysozyme, and by exposure to cell wall targeting antibiotics (e.g. β‐lactams), to which they are completely resistant. This is important because cell wall targeting antibiotics are the mainstay of modern infection therapy (Care, 2021) and because biofilms (Special states: biofilms) and multi‐drug‐resistant infections, against which such antibiotics often fail, are key indications for phage therapy. Therefore, targeting L‐forms with phages may be an important therapeutic option. However, L‐form susceptibility to phages has not yet been well characterized except for a few reports, suggesting that the capacity for efficient lysis of L‐forms is retained at least by some phages (Kawacka *et al*, 2020).

### Special states: intracellular pathogens

**Figure 5:**
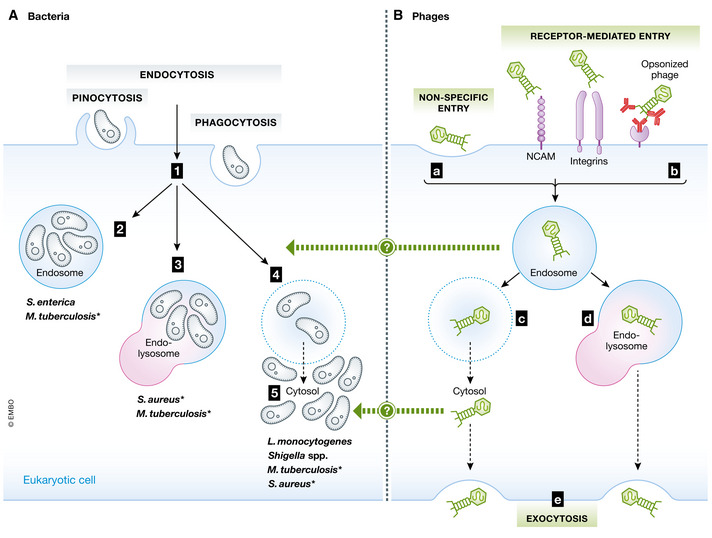
Intracellular lifestyles of bacterial pathogens and barriers in the treatment of intracellular infections (A) Intracellular bacteria penetrate mammalian cells by endocytosis, phagocytosis or pinocytosis (1), and reside inside different subcellular compartments: the endosome (2), the endolysosome (3; formed by fusion of the endosome with a lysosome) or the cytosol (5), after escaping endosome inclusion (4, light‐blue dotted ring) (Cossart & Sansonetti, 2004; Ray *et al*, 2009). (B) Free phages must cross the eukaryotic cell membrane by non‐specific (a) or receptor‐mediated (b) entry. Endocytosed phages may then escape the vacuole (c, light‐blue dotted ring) or remain in the endosome (blue ring) with eventual fusion to a lysosome (d). After cytosolic release (c) or lysosomal fusion (d), viable phages may be released from the mammalian cell via exocytosis (e). * indicates bacteria that can replicate in more than one host cell compartment. The exact details of how phages reach their intracellular targets are still largely unknown (?).

Certain bacterial pathogens responsible for high rates of infection and mortality (GBD Tuberculosis, 2018; Khalil *et al*, 2018; GBD Non‐Typhoidal *Salmonella*, 2019; GBD Antimicrobial Resistance, 2022) routinely replicate inside human cells including professional phagocytes such as monocyte‐derived macrophages (Ogawa & Sasakawa, 2006) (Fig 5A). These bacteria are protected from the immune system and from bactericidal agents in their intracellular niches. In addition, intracellular bacteria can take advantage of the biology of the host cell to disseminate to tissues beyond the site of infection. Most antibiotics commonly used in medicine do not penetrate mammalian cells efficiently and are therefore ineffective against intracellular pathogens (Abed & Couvreur, 2014; Kamaruzzaman *et al*, 2017). The few exceptions (e.g. quinolones, macrolides and tetracyclines) (Carryn *et al*, 2003; Kamaruzzaman *et al*, 2017) are widely used orally, and resistance to these is rising in target pathogens (WHO, 2017). Phages could therefore be of value for the treatment of intracellular infections.

The first evidence of phages crossing the eukaryotic cell barrier dates back more than 50 years (Monsur *et al*, 1970), and it is known that these viruses can penetrate human cells and even enter their nucleus (Nieth *et al*, 2015; Lehti *et al*, 2017; Nguyen *et al*, 2017; Zhang *et al*, 2017; Sweere *et al*, 2019). Phages may enter the eukaryotic cell non‐specifically by phagocytosis or pinocytosis, or through receptor‐mediated entry by binding specifically to cell surface structures like the neural cell adhesion molecule (NCAM; a major polysialic acid carrier that mimics bacterial receptors) or to cell surface integrins, or by antibody‐mediated uptake when phages are opsonized by circulating immunoglobulins (Bodner *et al*, 2021; Goswami *et al*, 2021) (Fig 5B).

Phages have been detected in early endosomes, endolysosomes and the Golgi apparatus (Nieth *et al*, 2015; Lehti *et al*, 2017; Zhang *et al*, 2017; Moller‐Olsen *et al*, 2018), and can escape eukaryotic vacuoles to reach bacteria replicating in the cytosol (Nieth *et al*, 2015). Phage–bacterium interactions in subcellular compartments are expected to be heavily modulated by the host eukaryotic cell, potentially in ways that alter phage infectivity or bacterial susceptibility as bacteria respond to intracellular stress (e.g. low pH, reactive oxygen species and antimicrobial peptides) and to nutrient depravation. Phages can certainly kill intracellular bacteria (Zhang *et al*, 2017; Moller‐Olsen *et al*, 2018), but further investigation of how phages reach their intracellular targets will be essential for designing successful therapeutic protocols.

### Special states: biofilms

In many natural niches, including human body sites (e.g. respiratory and urinary tract), both Gram‐positive and Gram‐negative bacteria live in complex sessile biofilm communities (Hall‐Stoodley *et al*, 2004), often polymicrobial. Bacterial biofilms are common in chronic and persistent infections (Bjarnsholt, 2013) and on abiotic surfaces such as medical devices (prosthetic joints, catheters, heart valves) (Donlan, 2001; Petrovic Fabijan *et al*, 2019). Diverse components (Smirnova *et al*, 2010) make up an extracellular matrix in which bacteria are embedded, which gives stability and strength to the growing biofilm (Flemming & Wingender, 2010). Biofilm formation and maturation are guided by the coordinated activity of embedded bacteria, regulated by refined quorum‐sensing mechanisms in response to population density variation (Parsek & Greenberg, 2005; Nadell *et al*, 2008). Bacteria in a biofilm exhibit different metabolic activity and physiological state depending on their position in the biofilm and on the age of the biofilm (Stewart & Franklin, 2008). Antibiotics are often ineffective against biofilm‐mediated infections as bacteria are physically protected from external agents and more tolerant to antimicrobial challenge due to their modified metabolism and often reduced growth states (Lebeaux *et al*, 2014; Yan & Bassler, 2019).

The finger‐like bacterial fimbriae and other adhesins that are important in biofilm initiation (Déziel *et al*, 2001; Pohlschroder & Esquivel, 2015; Maldarelli *et al*, 2016; Delerue & Ramamurthi, 2021) are also common phage receptors (Phage tail machines as sophisticated infection devices). Phages have proven useful against bacteria in biofilms (Abedon, 2019; Patey *et al*, 2019; Melo *et al*, 2020b; Petrovic Fabijan *et al*, 2021), but the study of these systems is difficult (Abedon *et al*, 2021b; Pires *et al*, 2021). Although phages often exhibit potent *in vitro* activity against bacteria in biofilms, effective biofilm eradication may require combination strategies (Verma *et al*, 2009; Seth *et al*, 2013; Tkhilaishvili *et al*, 2018; Henriksen *et al*, 2019; Morris *et al*, 2019), with failures attributed to difficulties in accessing target cells and the development of phage‐resistant subpopulations.

The biofilm matrix shields bacteria from phage attack by trapping phage particles and preventing diffusion (Sutherland *et al*, 2004; González *et al*, 2018; Dunsing *et al*, 2019; Melo *et al*, 2020b), and phage size and concentration have been shown to differentially impact biofilm disruption ability (González *et al*, 2018). A biofilm can protect phages from the eukaryotic immune system, and these trapped viruses may in turn limit biofilm growth (Simmons *et al*, 2018; Hansen *et al*, 2019; Bond *et al*, 2021) so that in a stabilized biofilm, bacteria and phages may coexist in dynamic equilibrium (Fernández *et al*, 2018; Hansen *et al*, 2019; Pires *et al*, 2021). Bacteria may produce matrix‐degrading substances when challenged with phages (Alcock & Palmer, 2021; de Cássia Oliveira *et al*, 2021) and can also secrete phage‐inactivating substances (Pires *et al*, 2021). *E. coli* can halt phage invasion of mature biofilms through expression of curli fibres that affect biofilm architecture, hinder phage diffusion and physically protect the bacterial cell surface (Price & Chapman, 2018; Vidakovic *et al*, 2018; Bond *et al*, 2021). Also relevant when considering phage therapy for chronic infections (Pires *et al*, 2017) is the fact that older biofilms are often characterized by thicker matrix and by subpopulations of bacteria that are less metabolically active (Testa *et al*, 2019), these two factors alone mitigating the potential impact of phage therapeutic intervention.

Phage‐produced lysins and depolymerases (Attachment mechanisms and receptor specificity) are less sensitive to biofilm heterogeneity, bacterial metabolic state and physical barriers and may have a role in matrix degradation (Olsen *et al*, 2018; Wu *et al*, 2019; Rakov *et al*, 2021; Shahed‐Al‐Mahmud *et al*, 2021). Delivery of phages or their derived enzymes together with antibiotics and/or disinfectants may be synergistic, with disruption of the extracellular matrix by phage enzymes and/or chemical antimicrobials expected to allow better access to subsequent antibiotics and phages (Chan & Abedon, 2015; Ferriol‐González & Domingo‐Calap, 2020).

Bacteria in biofilms use much the same adaptation mechanisms as free‐living bacteria (Phage–bacterium co‐adaptation) (Azeredo *et al*, 2021). Added protection derived from the population density in biofilms comes from quorum‐sensing signalling to manage receptor modulation (Moreau *et al*, 2017; Azeredo *et al*, 2021; León‐Félix & Villicaña, 2021), e.g. in *E. coli* (Høyland‐Kroghsbo *et al*, 2013) and *P. aeruginosa* (Høyland‐Kroghsbo *et al*, 2017; Broniewski *et al*, 2021), and through modification of bacterial physiology (Qin *et al*, 2017).

### The eukaryotic host: phage‐induced immune responses

The natural immunogenicity of phages may result in both an innate immune response (Petrovic Fabijan *et al*, 2020b; Khatami *et al*, 2021) and an adaptive immune response (e.g. phage‐specific antibodies) to viral nucleic acids (DNA or RNA) and proteins (capsid and tail) (Gonzalez‐Mora *et al*, 2020). The sustained phage viraemia arising from therapeutic infusion (Dąbrowska & Abedon, 2019; Petrovic Fabijan *et al*, 2020b) does not seem to present a safety risk but may be associated with modulation of the human immune response (Górski *et al*, 2017b; Petrovic Fabijan *et al*, 2020b; Khatami *et al*, 2021) by mechanisms that are as yet unclear. This topic has been well reviewed (Popescu *et al*, 2021), but key aspects to highlight include the following:

#### Phagocytosis

Non‐specific phagocytosis of viral particles may play a major role in the rapid clearance or neutralization of phages through the mammalian host reticuloendothelial system (Merril *et al*, 1996) and promote the presentation of antigens to T cells for the development of specific or adaptive immune response against phages themselves (Dąbrowska & Abedon, 2019). Phage binding may also facilitate phagocytosis of bacteria by macrophages or dendritic cells. Early studies (D'Herelle, 1923; Nelson, 1928) showed that phage‐resistant bacteria are protected from this effect, and it has been suggested that this “opsonization” process may be important for the eradication of pathogenic bacteria *in vivo* (Górski *et al*, 2017b) and may explain observations of reduced phage efficacy in neutropenic hosts (Roach *et al*, 2017).

#### Inflammation

Minor pro‐inflammatory responses *ex vivo* (Van Belleghem *et al*, 2017) and in treated patients (Khatami *et al*, 2021) have been attributed to LPS release into the system following bacterial lysis. However, the use of highly purified therapeutic phage preparations has not been associated with significant inflammatory responses (Górski *et al*, 2012; Krut & Bekeredjian‐Ding, 2018) so it is thought that contaminating endotoxins in early therapeutic phage preparations may have been primarily responsible for activation of Toll‐like receptor (TLR) signalling pathways and early reports of post‐infusion fevers (D'Herelle, 1930; Hashiguchi *et al*, 2010; Krut & Bekeredjian‐Ding, 2018).

#### Anti‐inflammatory immune response

Highly purified (“GMP‐grade”) phage preparations may induce the expression of key anti‐inflammatory genes, including IL‐1RA and IL‐10 family cytokines (Van Belleghem *et al*, 2017). An apparent anti‐inflammatory profile has been demonstrated both *in vivo* (Van Belleghem *et al*, 2017) and *in vitro* (Dhungana *et al*, 2021) and observed in critically ill patients with infective endocarditis and sepsis receiving adjunct phage therapy (Petrovic Fabijan *et al*, 2020b; Khatami *et al*, 2021). Other studies have shown a significant decrease in C‐reactive protein values, erythrocyte sedimentation rates and white cell counts in patients treated with phage (Miedzybrodzki *et al*, 2009), although these could equally be simple responses to reduced bacterial burden. It is conceivable that phages evolved to attack human colonizers and pathogens might also be able to survive attack by the immune system, and while the immunomodulatory and anti‐inflammatory mechanisms remain unclear, some studies suggest that phage interaction with immune cells may also be directly implicated (Górski *et al*, 2017a; Sweere *et al*, 2019).

#### Antiviral immune response

This has been well described in filamentous phages (Sweere *et al*, 2019). Pf phages can trigger maladaptive innate viral responses via TLR3 and interferon‐β production, and inhibition of TNF and phagocytosis, impairing bacterial clearance. It remains unclear, however, whether widely used therapeutic tailed phages can trigger similar antiviral responses.

#### Adaptive humoral immune response

Due to their immunogenic nature, phages can induce a strong humoral response (phage‐neutralizing IgG, IgM and, to a lesser extent, IgA antibodies), which can impact phage bioavailability *in vivo* and potentially hamper therapeutic success. The timing and strength of the humoral antiphage immune response mainly depend on phage immunogenic properties based on different structural protein composition (e.g. capsid proteins are known to be highly antigenic, for example the major capsid protein and outer capsid protein (Hoc) in T4‐like phages (Dąbrowska *et al*, 2014)), but are also affected by the route of administration, dose and the patient's immune status (Zaczek *et al*, 2016; Lusiak‐Szelachowska *et al*, 2017). Previous reports indicated that orally administered phages induce no or very weak humoral response in healthy volunteers (Sarker *et al*, 2012). In contrast, intravenously administered phages induce a strong humoral response, which usually arises within 10 days of phage therapy initiation (Pescovitz *et al*, 2011; Lusiak‐Szelachowska *et al*, 2014; Petrovic Fabijan *et al*, 2020b), with strong IgM induction in the first days of therapy, and high IgG levels recorded between 7 and 14 days. While earlier studies from the Hirszfeld Institute for Experimental Therapy (Poland) and the Eliava Institute (Georgia) showed no significant correlation between clinical outcome and level of antiphage antibodies (Lusiak‐Szelachowska *et al*, 2014), recent reports indicate that robust antibody response against certain phage types may limit phage efficacy *in vivo* and lead to therapeutic failure (Dedrick *et al*, 2021). Although our understanding of the influence of the humoral immune response on phage bioavailability and therapeutic success is limited, genetic engineering approaches (e.g. modification of phage capsid proteins) may prove key to overcoming these immunogenicity barriers (Hodyra‐Stefaniak *et al*, 2020).

Phages that have evolved to protect their prey populations by down‐regulating the host immune response may prove to be difficult choices in therapy. Conversely, phage‐mediated immunomodulation may be a good therapeutic trade‐off in severe sepsis where attenuation of a lethal cytokine‐mediated inflammatory response may be the most important therapeutic goal.

## Concluding remarks

In this review, we sought to highlight the main areas of phage and bacterial biology that may directly relate to therapeutic outcome and in need of further investigation (Table 1).

**Table 1:** Key biological aspects in phage–bacterium interaction that may affect clinical outcomes.

Biological mechanism	Biological role	Desired properties for therapy	Implications for therapy	Focus for improvement of clinical outcomes
Phage attachment	Infectivity (lytic activity)	High lytic activity: large burst size	Dosing and timing of administration	Diverse banks of characterized phages; genome engineering
Receptor specificity	Infectivity (lytic activity; host range)	Defined host range	Targeting; clinical spectrum of activity (target bacteria); resistance	Personalized therapy; curated phage/bacteria banks; AI/machine learning approaches; phage cocktails; phage “training”; genome engineering
Phage life cycle	Infectivity (lytic activity); transduction	High lytic activity; low transduction rates	Bacterial killing efficiency; transmission of virulence/resistance	Phage genomics; curated phage banks; genome engineering
Bacterial cell physiological state/ density	Niche colonization and invasion	High lytic activity; high penetration	Dosing and timing of administration; phage/antibiotic synergy; target diseases	Smart delivery
Bacterial lifestyle	Communal (biofilms); intracellular	High penetration	Penetration (target availability); clinical spectrum of activity (type of disease)	Smart delivery
Co‐adaptation	Microbial evolution	Poor ability to elicit resistance; stable high infectivity	Resistance development	Phage–phage and phage–antibiotic synergy

However, bringing phages into the pharmacopoeia requires attention to several other areas that we have not fully discussed. The limited host range of most therapeutic phages means that this precision therapy needs well‐curated and accessible phage sources, which is a biobanking and information management challenge (Nagel *et al*, 2022). The prioritization of target infections is key in determining the content and purpose of such collections and will vary with the intended use and the balance of research and commercial sustainability agendas (commercial priorities in sustainable phage production will differ from research priorities).

Modification of phages to enhance their therapeutic potential (Pires *et al*, 2016; Brown *et al*, 2017; Chen *et al,* 2019; Kilcher & Loessner, 2019; Monteiro *et al*, 2019) is complicated by the presence of large proportions of uncharacterized genetic material (“dark matter”) in phage genomes, which must be experimentally addressed (Hatfull & Hendrix, 2011; Wittebole *et al*, 2014; Hatfull, 2015; Philipson *et al*, 2018; Moreno‐Gallego & Reyes, 2021).

The complexities of variable penetration into eukaryotic cells, tissue layers and mammalian host compartments such as the gut have also not been addressed in this review, but readers are referred to others for this important topic (Barr *et al*, 2015; Dąbrowska & Abedon, 2019; Hofer, 2019; Huh *et al*, 2019). We have also set aside the difficulties of production and manufacturing protocols for GMP‐grade phage preparations: safe phage therapy involves not only quality processing but also the careful selection of suitable production hosts to ensure efficiency and avoid inadvertent gene transduction. The ideal phages for formulation into therapy must not only behave predictably in complex microbial niches but must also be readily purified and stable in storage (Merabishvili *et al*, 2018; Moelling *et al*, 2018; Rohde *et al*, 2018; Pirnay *et al*, 2019). The safety of phages for compassionate use means that there may be some opportunities to “learn as we go”, but we must now proceed with eyes wide open, and we must be guided as much as possible by the basic physiology of the main actors, the phages and their bacterial hosts.

## Author contributions

**Carola Venturini:** Conceptualization; Visualization; Writing—original draft; Writing—review & editing. **Aleksandra Petrovic Fabijan:** Conceptualization; Visualization; Writing—original draft; Writing—review & editing. **Alicia Fajardo Lubian:** Conceptualization; Visualization; Writing—original draft; Writing—review & editing. **Stefanie Barbirz:** Conceptualization; Visualization; Writing—original draft; Writing—review & editing. **Jonathan Iredell:** Conceptualization; Writing—review & editing.

In addition to the CRediT author contributions listed above, the contributions in detail are:

CV, JI, APF, AFL and SB conceptualized and wrote the manuscript. CV coordinated the preparation of the manuscript. CV and JI made final edits to the manuscript.

**Pending issues**

- Limited well‐curated and accessible phage biobanks
- Narrow host range
- Exclusive reliability on obligate lytic phages
- Occurrence of phage‐resistant bacterial mutants
- Priority types of infection targeted
- Application of phage cocktails vs monophage therapy
- Therapeutic phage monitoring, dosing and administration protocols
- Formulation and stabilization of phage therapeutics
- Regulatory and intellectual property protection

## Disclosure and competing interests statement

The authors declare that they have no conflict of interest.

## For more information

Online links to relevant sources

- International Society for Viruses of Microorganisms (ISVM) (international non‐profit organization dedicated to the advancement of the science and utility of the viruses of microorganisms, including archaeal viruses, bacteriophages and the viruses of microbial eukaryotes)—http://www.isvm.org/
- Phage Directory (curated database of phage laboratories, phages and host strains to advance research and phage therapy)—https://phage.directory/
- Phages for Human Applications Group Europe (international non‐profit organization to support phage research and phage therapy in Europe)—P.H.A.G.E. vzw ‐ Home (p‐h‐a‐g‐e.org)
- Phage Australia (Australian national network of phage researchers and clinician‐scientists to professionalize phage therapy)—https://phageaustralia.org/
- Center for Phage Biology and Therapy at Yale (newly established centre to advance phage biology and develop phage therapy into a safe, effective, scientifically sound and rational approach to infection control)—http://www.yalephagecenter.com/
- Centre on Innovative Phage Applications and Therapeutics (first dedicated phage therapy centre in North America)—Center for Innovative Phage Applications and Therapeutics (ucsd.edu)
